# Supplementary material for: A systematic review of whole disease models for informing healthcare resource allocation decisions
Source: PLoS One. 2023 Sep 14;18(9):e0291366. doi: 10.1371/journal.pone.0291366 (PMC10501624; doi:10.1371/journal.pone.0291366)
Supplement: S1 Text — (DOCX) [file pone.0291366.s001.docx]

**S1 Table. Electronic search strategies**

**1.1 MEDLINE search strategy**

Database used: Ovid MEDLINE(R) ALL

1946 to July 18, 2022

|  | **Search terms** | **Results** |
| --- | --- | --- |
| 1 | ((whole or full or entire or comprehensive) adj3 (system$ or pathway$ or care or treat* or guideline$ or simulat* or model* or disease$)).mp. | 110341 |
| 2 | (prevent* adj7 (treat* or therap*)).ti,ab. | 277880 |
| 3 | (upstream adj2 downstream).ti,ab. | 6037 |
| 4 | ((service or pathway) adj2 (change* or configuration$)).ti,ab. | 2909 |
| 5 | (with adj2 without).ti,ab. | 456363 |
| 6 | 1 or 2 or 3 or 4 or 5 | 843146 |
| 7 | exp Cost-Benefit Analysis/ | 90325 |
| 8 | (cost$ adj2 (effect* or benefit$ or utility or utilities or outcome$ or consequence$)).mp. | 232553 |
| 9 | (cost$ adj2 minimi*).mp. | 4830 |
| 10 | 7 or 8 or 9 | 236128 |
| 11 | exp Decision Theory/ or exp Decision Making, Computer-Assisted/ or Decision Support Systems, Management/ or exp Decision Making/ or Decision Support Systems, Clinical/ or Decision Trees/ or Decision Making, Organizational/ or exp Decision Support Techniques/ | 459804 |
| 12 | Computer Simulation/ or Patient Simulation/ or models, theoretical/ or exp models, organizational/ or exp models, statistical/ or exp models, economic/ or monte carlo method/ or Markov Chains/ | 807358 |
| 13 | (decision adj (tree$ or analysis or analyses or analytic$ or support)).mp. | 68202 |
| 14 | ((disease or mathematical or optimization or optimisation or decision$ or economic$ or pharmacoeconomic$ or simulation or cohort or Markov or state$transition or patient$level or individual$level or individual sampling or event history or agent$based) adj model*).ti,ab. | 106859 |
| 15 | ((discrete event or discrete individual or agent based or hybrid or inverse or monte carlo or real time) adj simulation).ti,ab. | 13592 |
| 16 | (system dynamics or DES).ti,ab. | 57923 |
| 17 | 11 or 12 or 13 or 14 or 15 or 16 | 1356098 |
| 18 | 6 and 10 and 17 | 2820 |
| 19 | (letter or news or editorial or historical article).pt. | 2357035 |
| 20 | 18 not 19 | 2811 |
| 21 | exp animals/ not humans/ | 5031505 |
| 22 | 20 not 21 | 2760 |
| 23 | limit 22 to english language | 2683 |

**1.2 EMBASE search strategy**

Database used: Embase 1974 to 2022 July 18

|  | **Search terms** | **Results** |
| --- | --- | --- |
| 1 | ((whole or full or entire or comprehensive) adj3 (system$ or pathway$ or care or treat* or guideline$ or simulat* or model* or disease$)).mp. | 141063 |
| 2 | (prevent* adj7 (treat* or therap*)).ti,ab. | 380096 |
| 3 | (upstream adj2 downstream).ti,ab. | 7244 |
| 4 | ((service or pathway) adj2 (change* or configuration$)).ti,ab. | 4142 |
| 5 | (with adj2 without).ti,ab. | 651237 |
| 6 | 1 or 2 or 3 or 4 or 5 | 1167321 |
| 7 | *economic evaluation/ or exp "cost benefit analysis"/ or exp "cost effectiveness analysis"/ or exp "cost minimization analysis"/ or exp "cost utility analysis"/ | 258759 |
| 8 | (cost* adj2 (effect* or benefit* or utility or utilities or outcome* or consequence*)).mp. | 391336 |
| 9 | (cost* adj2 minimi*).mp. | 8827 |
| 10 | 7 or 8 or 9 | 399119 |
| 11 | exp decision support system/ or decision making/ or "decision tree"/ or clinical decision making/ or decision theory/ or medical decision making/ | 451872 |
| 12 | exp simulation/ or computer model/ or individual based population model/ or population model/ or exp mathematical model/ or stochastic model/ or exp disease model/ or hidden Markov model/ or statistical model/ | 1424867 |
| 13 | (decision adj (tree* or analysis or analyses or analytic* or support)).mp. | 73722 |
| 14 | ((disease or mathematical or optimization or optimisation or decision* or economic* or pharmacoeconomic or simulation or cohort or Markov or Markov chain or state transition or patient level or individual level or individual sampling or event history or agent based) adj model*).ti,ab. | 139067 |
| 15 | ((discrete event or discrete individual or agent based or hybrid or inverse or monte carlo or real time) adj simulation).ti,ab. | 15574 |
| 16 | (system dynamics or DES).ti,ab. | 48359 |
| 17 | 11 or 12 or 13 or 14 or 15 or 16 | 1977335 |
| 18 | 6 and 10 and 17 | 4615 |
| 19 | (letter or editorial or note).pt. | 2866280 |
| 20 | 18 not 19 | 4594 |
| 21 | limit 20 to (human and english language) | 3943 |

**1.3 NHS Economic Evaluation Database (NHSEED) and the Health Technology Assessment Database (HTA) search strategy**

Database used: NHSEED and HTA accessed via Cochrane library interface (<http://onlinelibrary.wiley.com/cochranelibrary/search>) on 19/July/2022

|  | **Search terms** | **Results** |
| --- | --- | --- |
| 1 | ((whole or full or entire or comprehensive) adj3 (system* or pathway* or care or treat* or guideline* or simulat* or model* or disease*)) IN NHSEED, HTA | 358 |
| 2 | (prevent* adj7 (treat* or therap*)) IN NHSEED, HTA | 1,533 |
| 3 | (upstream adj2 downstream) IN NHSEED, HTA | 0 |
| 4 | ((service or pathway) adj2 (change* or configuration*)) IN NHSEED, HTA | 15 |
| 5 | (with adj2 without) IN NHSEED, HTA | 1,189 |
| 6 | #1 OR #2 OR #3 OR #4 OR #5 | 2,932 |
| 7 | MeSH DESCRIPTOR Decision Theory EXPLODE ALL TREES | 873 |
| 8 | MeSH DESCRIPTOR Decision Making EXPLODE ALL TREES | 447 |
| 9 | MeSH DESCRIPTOR Decision Trees EXPLODE ALL TREES | 864 |
| 10 | MeSH DESCRIPTOR Decision Support Techniques EXPLODE ALL TREES | 1,629 |
| 11 | MeSH DESCRIPTOR Computer Simulation EXPLODE ALL TREES | 494 |
| 12 | MeSH DESCRIPTOR Patient Simulation EXPLODE ALL TREES | 21 |
| 13 | MeSH DESCRIPTOR Models, Organizational EXPLODE ALL TREES | 105 |
| 14 | MeSH DESCRIPTOR Monte Carlo Method EXPLODE ALL TREES | 427 |
| 15 | MeSH DESCRIPTOR Markov Chains EXPLODE ALL TREES | 2,056 |
| 16 | (decision adj (tree* or analysis or analyses or analytic* or support)) IN NHSEED, HTA | 3,610 |
| 17 | ((disease or mathematical or optimization or optimisation or decision* or economic* or pharmacoeconomic* or simulation or cohort or Markov or state*transition or patient*level or individual*level or individual sampling or event history or agent*based) adj model*) IN NHSEED, HTA | 5,665 |
| 18 | ((discrete event or discrete individual or agent based or hybrid or inverse or monte carlo or real time) adj simulation) IN NHSEED, HTA | 856 |
| 19 | (system dynamics or DES) IN NHSEED, HTA | 627 |
| 20 | #7 OR #8 OR #9 OR #10 OR #11 OR #12 OR #13 OR #14 OR #15 OR #16 OR #17 OR #18 OR #19 | 8,652 |
| 21 | MeSH DESCRIPTOR Cost-Benefit Analysis EXPLODE ALL TREES | 13,213 |
| 22 | (cost* adj2 (effect* or benefit* or utility or utilities or outcome* or consequence*)) IN NHSEED, HTA | 17,798 |
| 23 | (cost* adj2 minimi*) IN NHSEED, HTA | 642 |
| 24 | #21 OR #22 OR #23 | 18,516 |
| 25 | #6 AND #20 AND #24 | 1,316 |
