# Supplementary material for: A systematic review of whole disease models for informing healthcare resource allocation decisions
Source: PLoS One. 2023 Sep 14;18(9):e0291366. doi: 10.1371/journal.pone.0291366 (PMC10501624; doi:10.1371/journal.pone.0291366)
Supplement: Attachment — Submitted filename: Rebuttal letter.docx [file pone.0291366.s007.docx]

**Editor’s comments**

While PLOS ONE does consider systematic reviews and meta-analyses for publication, we require that these submissions to provide a comprehensive review of the relevant literature and therefore be up-to-date within the last 6-12 months (http://www.plosone.org/static/guidelines#systematic). In the case of your submission, we are concerned that your search timeline ends in 21 July 2020 and is therefore not sufficiently comprehensive in its scope. As a result, we do not feel that your submission currently meets our internal standards for systematic review submissions.

If you choose to resubmit, we require that you update your Methods section to expand on the timelines of the search and consider the additional studies in your subsequent analyses. If you are able to address this concern through revisions to your manuscript, we may be willing to consider a resubmission for your work. The revised manuscript should be submitted as a new submission to PLOS ONE and accompanied by a cover letter that refers to the original submission number and outlines details of how the revisions have been completed.

**Authors’ response**

As advised by the editor, an update search was conducted on 18^th^ July 2022. 493 studies which were published between 21^st^ July 2020 and 18^th^ July 2022 were identified from the update search, of which six studies were found to meet our inclusion criteria. The entire manuscript, figures, and all supplementary materials have been updated throughout based on the findings of the update search. The updated texts in the manuscript were highlighted in yellow.
