# Supplementary material for: A systematic review of whole disease models for informing healthcare resource allocation decisions
Source: PLoS One. 2023 Sep 14;18(9):e0291366. doi: 10.1371/journal.pone.0291366 (PMC10501624; doi:10.1371/journal.pone.0291366)
Supplement: Attachment — Submitted filename: Response letter.docx [file pone.0291366.s008.docx]

**Reviewer #1**

This systematic review identified and summarized the characteristics of existing whole disease models (WDMs), and critically assessed their quality using the NICE appraisal checklist for economic evaluations. The information reported by this article can be used to help researchers, commissioners, or other stakeholders to rapidly locate relevant WDMs in the disease areas that they are interested in and critically appraise existing WDMs. Recommendations for future research can be used to fill evidence gaps and improve the quality and reporting of future WDMs. This article is well written, but I have the following concerns about the manuscript.

Response: Many thanks for recognising the importance of our work and taking your time to improve our manuscript.

1. The definition of “disease-level constrained maximisation of health decision rule” in this article was ambiguous, please use more straightforward words to clarify.

Response: As the reviewer suggested, an easier to understand definition of “disease-level constrained maximisation of health decision rule” has been added to the manuscript (p4, line 70-77):

“Under this decision rule (disease-level constrained maximisation of health decision rule), the optimal resource allocation for the whole care pathway can be identified by testing all possible combinations of service configurations (or in another word, all possible combinations of investment and disinvestment options) [9]. The service configuration which produces the greatest health benefits within the budget constraint of the service is considered to be most cost-effective.”

2. Lines 129-130, the subtitle of “Assessment of abstracts for inclusion” was inconsistent with the text of “Screening of abstracts and papers against the inclusion criteria...”. Please unify the descriptions.

Response: Thanks for spotting this inconsistency. The subtitle has been changed to “Assessment of abstracts and papers for inclusion” (p6, line 133).

3. Lines 203, “The characteristics of the remaining 26 WDMs...” should be “The characteristics of the remaining 32 WDMs...”.

Response: Thanks for spotting this mistake. The original sentence has been changed to “The characteristics of the remaining 32 WDMs….” (p9, line 207).

4. Table 2, the total number of items in the “Modelling techniques adopted” and “Modelling software” sections was not equal to the total number of included WDMs (n=43). This issue can also be found in the corresponding text (lines 212-216). Please check and correct them.

Response: Thanks for spotting this mistake. We have carefully checked the included studies and corrected that the number of studies for “Modelling techniques adopted” and “Modelling software” in both Table 2 (p19) and the text (p9, line 217-220).

5. Line 378, “the inter-reviewer agreement is moderate (Cohen's kappa = 0.56)” was inconsistent with the statement of “Cohen's kappa was 0.58” in the result section (line 180). Please double-check the figures.

Response: Thanks for spotting this mistake. The Cohen’s kappa in the Discussion section has been corrected to 0.58 (p27, line 423).

6. Lines 390-391, the data in the sentence “of the 37 WDMs included, only three demonstrated that they allow the use of disease-level constrained optimisation” needs to be updated to reflect “the update search”.

Response: Thanks for spotting this mistake. The number in the sentences have been updated to reflect new studies identified from the update search (p27, line 435-436): “Of the 43 WDMs included, only four [31, 49-51] demonstrated that they allow the use of disease-level constrained optimisation.”

7. It is suggested that the review further reflect on the impact of the development and application of WDMs on disease management and clinical pathway optimization, and then provide recommendations on the content and direction for future WDM construction.

Response: Thanks for your comment. As advised:

- a new section entitled ‘Use of WDMs in clinical guidelines’ has been added to the Discussion section (p24-25, line 350-371); and
- a new recommendation about the future research of WDM has been added to p26, line 390-406.

The newly added paragraphs can be found below.

**Use of WDMs in clinical guidelines** (p24-25, line 350-371)

“By covering the entire care pathway and most key interventions within a single modelling framework, whole disease modelling can be used as an ideal foundation for economic evaluations in clinical guidelines. So far, whole disease modelling has been piloted on three published NICE clinical guidelines, covering three different disease areas: colorectal cancer [88], prostate cancer [89] and atrial fibrillation [90]. The reported advantages of using whole disease modelling for the selected NICE guidelines are: (a) it produces a considerably larger amount of economic information compared to traditional ‘piecewise’ models; (b) it improves consistency of cost-effectiveness results by using a single analytic framework and a common set of assumptions and data sources; (c) it can be used to compare the cost-effectiveness of interventions at different parts of the pathway (e.g. prevention and treatment), and explore system interdependencies between different interventions; and (d) once developed, WDMs can be reused to consider other related questions or to incorporate new evidence [30, 32].

The reported disadvantages of using whole disease modelling are mainly related to the technical and practical barriers. Large and complex models like WDMs are likely to be more prone to verification (programming) errors, more difficult to validate, and more difficult to explain to decision-makers than simple models. As a result, the development of each WDM has been estimated to take at least 12 months’ time of a full-time modeller. However, as Professor Alan Williams [91] pointed out, if creating such large-scale models is horrendously complicated, it is because reality is horrendously complicated; the more complex the reality is, the more dangerous it is to rely on intuitive short-cuts rather than careful analyses. Therefore, ‘more complex areas require models that respect complexity’ [83]. Given that WDMs, once developed, can be adapted and reused to provide ongoing support for decision-making across the entire care pathway, in the long-term, the benefits of using whole disease modelling in the context of clinical guidelines may outweigh the initial investment of time and money.”

**New recommendations on future research of WDM** (p26, line 390-406)

“In addition to the recommendation listed above, future research exploring methods for reducing the development time of WDMs is required to encourage greater use of the whole disease modelling approach. There are two solutions which could potentially help to reduce the development time of a WDM. The first solution is to adopt a team approach. The development of a WDM can be divided into several interrelated but different task modules, each of which requires a different set of skills (e.g., development of a conceptual model, identification and preparation of relevant input data, implementing the conceptual model within a computer software, and communicating the model results to stakeholders). This means the development work for a WDM can be potentially assigned to more than one researcher working in parallel, which would accelerate the development time of a WDM. However, the adoption of a team approach will lead to increased labour costs. The second solution is to develop templates for whole disease modelling. There are some similarities in terms of building health economic models for diseases of the same or similar types. For example, the modelling of cancer usually involves tumour progression from early stages to late stages, while the modelling of mental health problems usually involves repeated transitions between remission and relapse. Even for patients with different types of diseases, their QALYs accumulated at a specific stage of the care pathway usually depend on similar factors, such as: patient’s starting age, end age, current disease status, comorbidities and/or adverse events of interventions. The code for implementing these common functions can be made into a modelling template (or different templates for different types of disease) and published online. These templates may help to reduce the development time of future WDMs.”

**Reviewer #2**

A systematic review of whole disease models for informing healthcare resource allocation decisions

There has been an increase in the number of WDMs since 2010. However, their quality is generally
low which means they may require significant modification before they could be re-used, such as modelling AEs of interventions and incorporation of PSA. Sufficient details of the WDMs need to be reported to allow future reuse/adaptation

**Query to the editor**: Underneath "Reviewer #2", we can only see three sentences, all of which were copied and pasted from the conclusion of our paper. So we assumed Reviewer 2 didn’t leave any specific comments for us to address. However, if this is not the case, please let us know. Thanks!
