# Supplementary material for: A systematic review of whole disease models for informing healthcare resource allocation decisions
Source: PLoS One. 2023 Sep 14;18(9):e0291366. doi: 10.1371/journal.pone.0291366 (PMC10501624; doi:10.1371/journal.pone.0291366)
Supplement: Attachment — Submitted filename: Response letter.docx [file pone.0291366.s009.docx]

Reviewer #3: I would like to express my sincere gratitude for the opportunity to review and learn from the work presented, which is of utmost scientific relevance.

In this regard, I would like to raise some questions that I believe could contribute to the improvement of the study methodology.

Response: Thank you for dedicating your time to review our paper, and we sincerely appreciate your thoughtful and constructive comments. We carefully considered each of your points and have provided responses below.

Firstly, the authors may consider updating their search strategy, as it appears that the searches were only conducted until June 2022.

Response: As advised by the reviewer, we conducted a second update search on 23^rd^ July 2023 and retrieved 285 additional records published since the first update search. One more whole disease model and two more pathway models were identified from the second update search. The manuscript, figures, tables and supplementary materials have all been updated throughout.

Secondly, I noticed that the exclusion of 29 studies was based on pathway models, despite the fact that PROSPERO allowed for their inclusion (https://www.crd.york.ac.uk/PROSPERO/display_record.php?RecordID=199875). It would be helpful if the authors could clarify the reason for this discrepancy.
Response: The reviewer is correct that our initial protocol allowed for inclusion of pathway models. However, during the course of conducting pilot searches and consulting with experts in the field, we realized that the number of whole disease models (WDMs) that met our pre-defined criteria might be limited. To ensure comprehensive coverage and enrich the findings of our study, we made the decision to expand the scope and include both WDMs and pathway models in our analysis.

Thanks for spotting the discrepancy. The focus of our review is always on whole disease models (WDMs). However, during the course of conducting pilot searches and consulting with experts in the field, we realised that the number of whole disease models (WDMs) that met our pre-defined criteria might be limited. To ensure comprehensive coverage and enrich the findings of our study, in the protocol, we made the decision to relax the criteria of a WDM by including pathway models, which are ‘semi-WDM’ that evaluate multiple decision points only for people who have already developed the disorder of interest. However, to our surprise, in the formal review, forty-three models were found to meet the full criteria of a WDM, of which thirty-one were published after Year 2010. This means we have enough WDMs to discuss in the main paper and no longer need to relax the inclusion criteria. It should be noted that although we decided to exclude pathway models from the main paper, we still extracted data from the identified pathway models in case they could be useful to some readers. The data extracted from pathway models can be found in S3 Table “Characteristics of included pathway models”.

Additionally, I would like to bring to the authors' attention that the methodology of a systematic review does not permit exclusion of studies based on language or geographic location without adequate justification. Thus, I suggest that the authors include studies in English, Portuguese, and Spanish in their analysis.
Response: We apologise for any misunderstanding in our manuscript. We confirm that our study did indeed exclude non-English papers. However, we would like to emphasise that no studies were excluded based on geographic locations, and the included studies represented a diverse range of countries, including more than twenty different nations.

As the reviewer correctly pointed out, the ideal approach in systematic reviews is to include all relevant studies regardless of language. We recognise the significance of this approach and would love to include as many languages as possible. However, our review was conducted on a voluntary basis without external funding or dedicated resources. The reviewing team members have been working on this review during their non-working hours, which constrained our capacity to handle studies in languages beyond English.

The Cochrane guidance and Centre for Reviews and Dissemination (CRD) guidance both acknowledge the considerable resources required to review papers in all languages. When it is not feasible to do so, they recommend acknowledging this limitation in the paper and discussing its potential implications. To align with these recommendations, we have included the following limitations and discussions in the paper:

- Line 427-428: “non-English literature and grey literature were not searched due to constraints in time, resources, and expertise within the reviewing team for non-English languages.”
- Line 431-433: “Since the aim of this review was to provide an overview of existing WDMs, rather than to synthesise the results of the identified studies, we reckon the negative consequences of missing relevant studies are relatively small.”

Moving forward, we will make efforts to secure additional resources and expertise to improve the inclusivity of our research and enhance the quality of future systematic reviews.

References:

- Higgins JPT, Thomas J, Chandler J, Cumpston M, Li T, Page MJ, Welch VA (editors). Cochrane Handbook for Systematic Reviews of Interventions version 6.3 (updated February 2022). Cochrane, 2022. Available from [www.training.cochrane.org/handbook](http://www.training.cochrane.org/handbook).
- Systematic Reviews. CRD's guidance for undertaking reviews in health care. Centre for Reviews and Dissemination, University of York, 2009.

Furthermore, I would appreciate a justification for the use of the National Institute for Health and Care Excellence (NICE) tool, which was replaced by the JBI tool.

Response: We considered eight checklists for the current review, including the JBI tool (Gomersall et al, 2015), the Drummond checklist (Drummond et al, 1996), the CHEERS checklist (Husereau et al, 2022), and other commonly used tools. We believe all the checklists that we considered are useful tools for assessing the quality of economic evaluations. Reviewers should choose the checklist that is most appropriate for the aims and design of their systematic review. The reason why we think the NICE checklist would be more appropriate for the purpose of our review is that our aim is to assess the methodological quality of the included models, rather than assessing the applicability of study findings to the decision problem of interest. The NICE checklist explicitly separates criteria for methodological quality from criteria for the applicability of results by having two different sections (Section 1 for assessing the applicability of results and Section 2 for assessing methodological quality), while the JBI tool combines these two types of criteria into one section. Therefore, we thought it would be more straightforward for us to apply the criteria of Section 2 of the NICE checklist to our included studies.

References:

1) Gomersall JS, Jadotte YT, Xue Y, Lockwood S, Riddle D, Preda A. Conducting systematic reviews of economic evaluations. Int J Evid Based Healthc. 2015;13(3):170–178.
2) Drummond M, F., Jefferson TO. Guidelines for authors and peer reviewers of economic submissions to the BMJ. British Medical Journal. 1996;313:275-83.
3) Husereau D, Drummond M, Augustovski F, de Bekker-Grob E, Briggs A H, Carswell C et al. Consolidated Health Economic Evaluation Reporting Standards 2022 (CHEERS 2022) statement: updated reporting guidance for health economic evaluations BMJ 2022; 376 :e067975 doi:10.1136/bmj-2021-067975
4) National Institute for Health and Care Excellence. Developing NICE guidelines: the manual. London, UK: National Institute for Health and Care Excellence; 2014.

Also, I am curious to know if the authors searched EMBASE with MESH terms, as this appears to be an incorrect approach.

Response: Thank you for raising this question. We understand the uniqueness of EMBASE's subject headings, which are often referred to as 'Emtree' but sometimes 'MeSH', depending on the searching platform used. In our study, we utilised the Ovid platform to conduct the electronic search, where the specific list of subject headings for EMBASE is termed 'MeSH'. It's essential to clarify that this 'MeSH' list is distinct from the MeSH terms used for searching in Medline.

To ensure accuracy and relevance in our search strategy, we meticulously manually selected each subject heading for EMBASE. We did not rely on a simple copy-and-paste approach of MeSH terms from Medline.

Moreover, could the authors kindly specify which tool or software was used for study extraction and management?

Response: In the originally submitted paper, we reported that we used Endnote X9.3.3 for managing reference (line 131) but did not report which software was used for data extraction. This information has been added to the manuscript (line 140-141):

“The following information was extracted using Microsoft Excel (Microsoft Corporation, Microsoft 365 subscription) from all included studies….”

Additionally, I found the definition of "disease-level constrained maximization of health decision rule" to be ambiguous, and I suggest that the authors clarify it using more straightforward language.

Response: We apologise for any confusion caused by the original definition. We have revised the definition as follows using more straightforward language (line 72-76):

“Under this decision rule (disease-level constrained maximization of health decision rule), different combinations of healthcare services (investment and disinvestment options) are tested [9]. The one that maximises health benefits while staying within the available budget is considered the most cost-effective choice.”

Lastly, I would strongly recommend that the authors evaluate the quality of evidence in their systematic review using the GRADE tool.

Response: Thank you for the valuable suggestion to utilise the GRADE tool (Brunetti et al., 2013) for evaluating the quality of evidence in our systematic review. We acknowledge the significance of the GRADE tool in assessing the certainty and applicability of results from economic evaluations, particularly when making recommendations for specific settings. It effectively considers items of resource use, rates the confidence in effect estimates, and valuates resource use in terms of costs for the intended setting.

However, we would like to clarify the primary objective of our systematic review, which is to assess the methodological quality of the identified economic models, rather than focusing on the specific setting or the uncertainty of the effect. In this context, the NICE checklist appears to be more suitable for our review compared to the GRADE tool.

Reference:

Brunetti M, Shemilt I, Pregno S, Vale K, Oxman AD, Lord J, Sisk J, Ruiz F, Hill S, Guyatt GH, Jaeschke R, Helfand M, Harbour R, Davoli M, Amato L, Liberati A, Schünemann HJ. GRADE guidelines: 10. Considering resource use and rating the quality of economic evidence. Journal of Clinical Epidemiology 2013; 66: 140-150. <https://doi.org/10.1016/j.jclinepi.2012.04.012>
